# Cell-free therapy for canine osteoarthritis: current evidence and prospects

**DOI:** 10.1080/01652176.2022.2145620

**Published:** 2022-11-17

**Authors:** Khan Sharun, Sathish Muthu, Pratheesh D. Mankuzhy, Abhijit M. Pawde, Vikash Chandra, Jose M. Lorenzo, Kuldeep Dhama, Amarpal, G. Taru Sharma

**Affiliations:** a Division of Surgery, ICAR-Indian Veterinary Research Institute, Bareilly, India; b Department of Biotechnology, School of Engineering and Technology, Sharda University, Greater Noida, India; c Orthopaedic Research Group, Coimbatore, India; d Department of Orthopedics, Government Medical College and Hospital, Dindigul, India; e Department of Physiology, Kerala Veterinary and Animal Sciences University, Pookode, India; f Division of Physiology and Climatology, ICAR-Indian Veterinary Research Institute, Bareilly, India; g Centro Tecnológico de la Carne de Galicia, Adva. Galicia n° 4, Parque Tecnológico de Galicia, Ourense, Spain; h Área de Tecnoloxía dos Alimentos, Facultade de Ciencias de Ourense, Universidade de Vigo, Ourense, Spain; i Division of Pathology, ICAR-Indian Veterinary Research Institute, Bareilly, India; j National Institute of Animal Biotechnology, Hyderabad, India

**Keywords:** Dog, canine, osteoarthritis, mesenchymal stem cells, cell-free therapy, stem cell therapy, extracellular vesicles

## Abstract

Osteoarthritis is a progressive degenerative disease affecting joints. It is associated with structural and functional changes that cause lameness and pain in dogs. Mesenchymal stem cells (MSCs) are considered an ideal therapeutic candidate for treating inflammatory musculoskeletal conditions due to their paracrine and immunomodulatory characteristics. They are delivered intravenously or as intra-articular injections for treating canine osteoarthritis. However, *ex vivo* studies have confirmed that the osteoarthritic synovial fluid is cytotoxic to cultured MSCs. Therefore, intra-articular transplantation of viable MSCs should be considered counterproductive since it minimizes cellular viability. Similarly, the intravenous administration of MSCs limits the therapeutic effects on the organ of interest since most of the administered cells get trapped in the lungs. Therefore, cell-free therapeutic strategies such as conditioned media and extracellular vesicles (EVs) can potentially become the future of MSC-based therapy in managing canine osteoarthritis. It overcomes the limitations of MSC-based therapy, such as tumor differentiation, immunogenicity, and pulmonary embolization, and has advantages like low immunogenicity and off-shelf availability. In addition, they eliminate problems such as low cell survival, transmission of infections, and unpredictable behavior of the transplanted MSCs, thereby acting as a safe alternative to cell-based therapeutics. However, very limited data is available on the efficacy and safety of cell-free therapy using MSCs for managing canine osteoarthritis. Therefore, large-scale, multicentric, randomized clinical controlled trials are required to establish the therapeutic efficacy and safety of MSC-based cell-free therapy in clinical cases of canine osteoarthritis.

## 1. Introduction

Osteoarthritis, also called osteoarthrosis or degenerative joint disease, is a progressive degenerative disease affecting joints in both human and veterinary patients (Loeser et al. 2012; Anderson et al. 2020). It is characterized by loss and dysfunction of articular cartilage, joint capsule thickening, and new bone formation (osteophytes), resulting in limb dysfunction in animals (American College of Veterinary Surgeons 2022). It is reported that the prevalence of canine osteoarthritis is around 20% among the aged dog population (Anderson et al. 2020; Wright et al. 2022). Osteoarthritis in canines is associated with structural and functional changes that cause lameness and pain (Anderson et al. 2020). Inciting factors like elbow and coxofemoral joint dysplasia, patellar luxation, articular fractures, limb malformations, and cranial cruciate ligament disease contribute to the occurrence of canine osteoarthritis (Pye et al. 2022). The articular cartilage has a limited self-healing capacity as a terminally differentiated tissue (Gugjoo et al. 2019). The limited healing capacity can also be attributed to the inability of cells to migrate through the dense extracellular matrix (ECM) (Hunziker and Kapfinger 1998). In addition, the avascular, aneural, and alymphatic nature of the cartilage also contributes to the limited healing potential (Gugjoo et al. 2019; Sasaki et al. 2019). This indicates the need for exogenous therapeutic agents to facilitate the healing and regeneration of articular cartilage.

The current therapeutic strategies against osteoarthritis mainly focus on managing pain and inflammation (Brondeel et al. 2021). Therapeutic strategies for managing canine osteoarthritis include multimodal approaches consisting of either conservative, surgical, or a combination of both. It includes weight control, activity modification, rehabilitation (aqua therapy, acupuncture, and laser therapy), pain management, joint supplements (glucosamine sulfate, chondroitin sulfate, and omega-3-fatty-acids), and disease modulating agents (stem cells, platelet-rich plasma, and hyaluronic acid) (American College of Veterinary Surgeons 2022; Pye et al. 2022). Pain management strategies include the use of non-steroidal anti-inflammatory drugs (meloxicam, carprofen, deracoxib, etc.) or other adjunctive pain medications such as gabapentin, grapiprant, tramadol, anti-nerve growth factor monoclonal antibodies, and corticosteroids (American College of Veterinary Surgeons 2022; Pye et al. 2022).

In addition, several surgical procedures such as excision arthroplasty, prosthetic joint replacement, joint resurfacing, femoral head and neck excision, and arthrodesis (fusion of joints) are performed to restore mobility in dogs suffering from osteoarthritis (Johnson 2019). However, surgical treatment of osteoarthritis may not be appropriate or feasible for osteoarthritis in multiple joints (Johnson 2019). Therefore, non-surgical alternatives such as intra-articular and systemic medications utilizing regenerative therapeutics should be developed for managing canine osteoarthritis. Biological cell-based therapeutics such as platelet-rich plasma and expanded or non-expanded mesenchymal stem cells (MSCs) (stromal vascular fraction and bone marrow aspirate concentrate) have already been evaluated for treating joint disease in veterinary patients (Bogers 2018).

This review aims to establish the prospects of cell-free therapy in managing canine osteoarthritis and briefly introduce a less explored research area in canine medicine. However, the findings discussed in this paper are preliminary and limited due to insufficient clinical data indicating the need for large-scale, randomized controlled trials to establish the therapeutic potential of cell-free therapy in canine osteoarthritis.

## 2. Methodology

A literature search was performed using the Scopus database (https://www.scopus.com/) to investigate the current status of cell-free therapy in canine osteoarthritis with the following search strategy:

TITLE-ABS-KEY (((canine* OR dog*) AND ("conditioned medium*" OR "conditioned media*" OR exosome OR "extracellular vesicle*" OR microvesicle* OR secretome*) AND ("stem cell*") AND (osteoarthritis))). The search was limited to journal publications in English and excluded articles published in other languages. In addition, only original research articles were included, excluding books, book chapters, conference papers, reviews, editorials, letters, commentary, notes, perspectives, short surveys, erratum, and retracted papers.

## 3. Results

The search yielded only two research publications (Huňáková et al. 2020; Mocchi et al. 2021), indicating the scarcity of available evidence on using cell-free therapy in managing clinical cases of canine osteoarthritis. The characteristic features of these clinical studies are described in Table 1.

**Table 1.** Characteristics features of the clinical studies evaluating the therapeutic efficacy and safety of cell-free therapy in canine osteoarthritis.

Sl. no.	Treatment	Study population	Protocol	Outcome	Reference
1	Allogeneic adipose-derived MSC-conditioned medium.	Six Labrador retriever dogs with bilateral elbow osteoarthritis (No control group).	Intra-articular injection on days 0 and 14	Significant improvement in range of motion parameters after the treatment. Increases functional ability of dogs. No severe adverse events.	Huňáková et al. (2020)
2	Allogeneic freeze-dried powder containing canine adipose-derived MSCs-secretome resuspended in hyaluronic acid (Lyosecretome) in one joint and placebo (mannitol resuspended in hyaluronic acid) in the other joint (investigators and owners were blinded).	Three dogs (One Golden retriever and two Labrador) affected by naturally occurring bilateral knee or elbow osteoarthritis.	Intra-articular injection on days 0 and 40	Safe in dogs and does not induce any clinically significant local or systemic adverse response.	Mocchi et al. (2021)

## 4. Discussion

MSCs are considered as ideal therapeutic candidate for treating inflammatory musculoskeletal conditions due to their ability to interact with the inflammatory environment *via* paracrine and immunomodulatory mechanisms (Ivanovska et al. 2022). The available evidence indicates that intra-articular injection of MSCs is beneficial in managing canine osteoarthritis (Kriston-Pál et al. 2017; Bogers 2018; Olsen et al. 2019). MSCs isolated from different tissue sources (adipose tissue, bone marrow, umbilical cord, muscle, and synovial fluid) are used for cartilage regeneration (Sasaki et al. 2019). These stem cells ameliorate cartilage injury by secreting trophic factors, recruiting endogenous progenitor cells to the site of injury, and redirecting them to cartilage tissue differentiation (Sasaki et al. 2019; Domaniža et al. 2021). The therapeutic potential of MSCs is attributed to their anti-inflammatory, anti-apoptotic, anti-fibrotic, and immunomodulatory properties (Domaniža et al. 2021). With the advances in proteomics, the MSC secretome has been known to include bioactive signals, growth factors, and ECM molecules (Sze et al. 2007), where growth factors in the MSC secretome contribute to chondrogenesis (Domaniža et al. 2021). Histological analysis has also confirmed that the regenerated cartilage was of hyaline type following the intra-articular transplantation of allogeneic adipose-derived MSCs in dogs suffering from elbow osteoarthritis (Kriston-Pál et al. 2017). Among the MSCs derived from different tissue sources (bone marrow, inguinal adipose, infrapatellar fat pad, and synovial fluid), infrapatellar fat pad and synovium-derived MSCs exhibited better proliferation ability compared to others (Sasaki et al. 2018). However, synovium-derived MSCs exhibited the highest chondrogenic potential and are therefore considered ideal for canine cartilage regeneration applications (Sasaki et al. 2018).

MSCs can be delivered intravenously or as intra-articular injections for treating canine osteoarthritis (Kriston-Pál et al. 2017; Olsen et al. 2019). However, intra-articular administration of MSCs often requires sedation or general anesthesia. In addition, the procedure can be time-consuming in dogs with multiple osteoarthritic joints (Olsen et al. 2019). Moreover, *ex vivo* experiments have confirmed that the osteoarthritic synovial fluid is cytotoxic to cultured MSCs (Kiefer et al. 2015). Therefore, transplantation of viable MSCs into osteoarthritic joints should be considered counterproductive since it minimizes cellular viability. In accord, studies have confirmed that MSCs transplanted *via* intra-articular injection do not engraft into the endogenous cartilage to effect direct repair (Desando et al. 2013; Satué et al. 2019). MSCs were detected only in the medial meniscus and synovial membrane but not in the cartilage following the intra-articular injection of labeled adipose-derived MSCs into the stifle joints of rabbits with osteoarthritis (Desando et al. 2013). The therapeutic effect exhibited following the intra-articular injection of MSCs to osteoarthritic joints might be contributed mainly by the secretome (growth factors, chemokines, cytokines, and EVs) and activation of the resident progenitor cell population responsible for cartilage regeneration (Tofiño-Vian et al. 2018). Intravenous administration of allogenic adipose-derived MSCs was found to be well tolerated in dogs with naturally occurring elbow osteoarthritis (Olsen et al. 2019). However, pulmonary trapping will interfere with the transport of intravenously administered MSCs to the joint fluid limiting their therapeutic potential (Fischer et al. 2009; Olsen et al. 2019). In addition, adverse effects such as pulmonary edema and hemorrhage are reported following the intravenous injections of allogeneic bone marrow-derived MSCs in beagle dogs (Kang and Park 2014).

The therapeutic outcome of different clinical trials evaluating the efficacy of MSCs in canine osteoarthritis was also found to vary depending on the degree of inflammatory environment, animal models used (spontaneous or induced), dose and source of MSCs, route of administration, and inter-observer differences (applicable to subjective outcome parameters) (Bogers 2018; Brondeel et al. 2021). Moreover, the absence of regulatory standards or frameworks for MSC-based cellular therapy in veterinary regenerative medicine raises concerns regarding the production standards, safety, and clinical efficacy of commercially available cell-based products limiting their clinical applications (Ivanovska et al. 2022). Furthermore, the increasing number of commercial cell-based veterinary products that lack proper characterization and standardization contributes to poor product consistency (Whitworth and Banks 2014; Ivanovska et al. 2022). The premature commercialization of cell-based veterinary products without generating data from animal clinical trials could contribute to the disparity between purported benefits of cell based-therapeutics and their proven abilities (Whitworth and Banks 2014). Therefore, large-scale, multicentric, randomized controlled trials are required to better evaluate the therapeutic potential of cell-based veterinary products in clinical cases of canine osteoarthritis.

**Figure 1.**
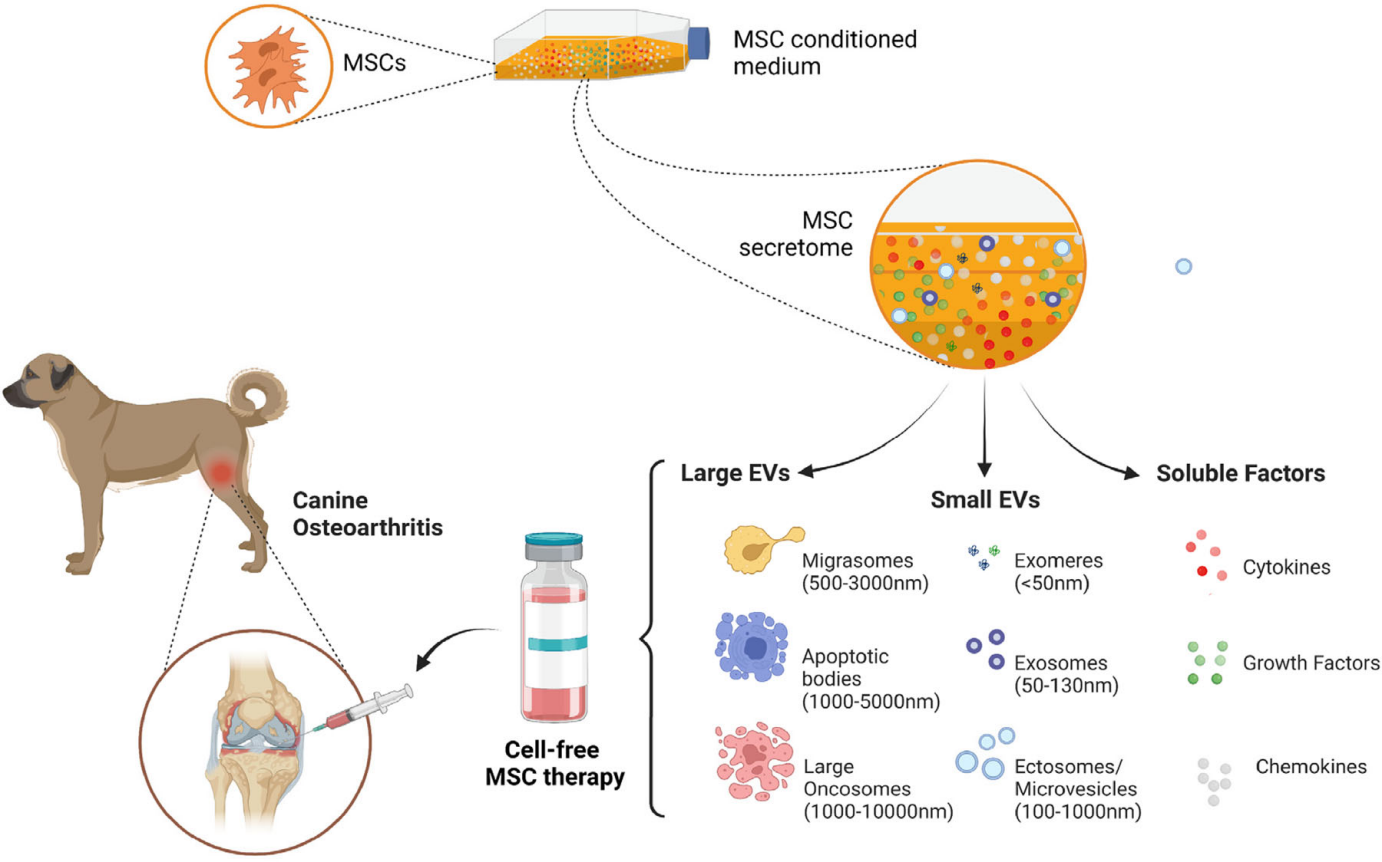
Illustrating the components that constitute the mesenchymal stem cell (MSC) derived secretome and the prospects of cell-free therapy in managing canine osteoarthritis. Diverse subtypes of extracellular vesicles (EVs) have already been defined. According to the International Society for Extracellular Vesicles (ISEV) recommendation, EVs are classified into small EVs (< 200 nm diameter) and large EVs (>200 nm) based on their size. MSCs – Mesenchymal Stem Cells (created using BioRender.com).

MSC-based cell-free strategies include using either the conditioned medium or different categories of membrane-bound extracellular vesicles (Sharun et al. 2022). MSC-derived conditioned medium contains several key biomolecules such as chemokines, cytokines, growth factors, and ECM components that accelerate the repair of injured tissues (Figure 1) (Huňáková et al. 2020). Besides that, MSCs also produce membrane-bound vesicles, including exosomes and microvesicles, that contribute to the therapeutic potential of the conditioned medium (Figure 1) (Huňáková et al. 2020; Sharun et al. 2022). EVs are broadly classified according to their size into exosomes, microvesicles, and apoptotic bodies (da Costa et al. 2021). Exosomes and microvesicles play a key role in mediating intercellular communication, whereas apoptotic bodies are produced during the disassembly of an apoptotic cell (Chandra and Sharma 2021; da Costa et al. 2021). Furthermore, nanosized EVs are classified into exomeres, large exosomes, small exosomes, and oncosomes. Recently, another category of EVs was identified, termed migrasomes. They are EVs that mediate migracytosis (da Costa et al. 2021).

Exosomes and microvesicles isolated from murine bone marrow-derived MSCs inhibited macrophage activation and protected chondrocytes from apoptosis during *in vitro* evaluation (Cosenza et al. 2017). Furthermore, the chondroprotective and anti-inflammatory properties of MSC-derived exosomes and microvesicles were further confirmed in the collagenase-induced osteoarthritis mice model (Cosenza et al. 2017). Similarly, intra-articular injection of MSCs and MSC-derived secretome produced similar results (early pain reduction and chondroprotective effect) and prevented cartilage damage in the collagenase-induced osteoarthritis mice model (Khatab et al. 2018). Therefore, the therapeutic effects produced by MSCs that protect mice from developing OA can also be generated by administering exosomes and microvesicles derived from them (Cosenza et al. 2017; Khatab et al. 2018). Exosomes isolated from adipose-derived MSCs upregulated the expression of the anti-inflammatory cytokine IL-10 and downregulated the pro-inflammatory markers such as IL-6, tumor necrosis factor-α (TNF- α), and nuclear factor kappa B (NF-κB) and when co-cultured with activated synovial fibroblasts (Zhao et al. 2020). Furthermore, their role in promoting chondrogenesis was confirmed due to the increased chondrogenic markers such as β-catenin and collagen type II (Zhao et al. 2020).

The efficacy of MSC-derived microvesicles was previously evaluated in a canine chondral defect model. Intra-articular injection of microvesicles isolated from bone marrow-derived MSCs contributed to the functional and morphological recovery of injured cartilage (Sabry et al. 2018). Conditioned medium isolated from adipose-derived MSC can be used to treat bilateral elbow joint osteoarthritis. Intra-articular injection of the allogeneic conditioned medium increased the functional ability of Labrador retriever dogs with elbow joint osteoarthritis (Huňáková et al. 2020). Similarly, freeze-dried MSC-secretome (lyosecretome) is another therapeutic strategy used to manage canine osteoarthritis (Mocchi et al. 2021). Intra-articular injection of lyosecretome in client-owned dogs with elbow or knee osteoarthritis was found to be safe and did not induce systemic or local adverse responses (Mocchi et al. 2021).

MSCs-derived exosome is a promising cell-free approach that delays the progression of osteoarthritis by inhibiting chondrocyte apoptosis, stimulating ECM secretion, promoting chondrocyte migration and proliferation, and maintaining chondrocyte homeostasis (Tao et al. 2017; Peláez et al. 2021). MSCs-derived exosomes function as the key messenger between the stem cells and injured tissues, operating almost similar to MSCs but lacking the disadvantages of cellular therapies (Joseph et al. 2020; Sharun et al. 2022). Although the preliminary findings indicate therapeutic utility in canine osteoarthritis, the actual role and efficacy of cell-free therapy in clinical cases are yet to be demonstrated.

The EVs released from the MSCs contain biologically active signaling molecules that can ameliorate the pathological progression of canine osteoarthritis (Pye et al. 2022). Furthermore, these MSC-derived exosomes include many proteins, lipids, ribonucleic acid, and deoxyribonucleic acid that modulate homeostasis and facilitate endogenous repair and regeneration (Jeyaraman et al. 2021; Zeng et al. 2022). Most of these proteins are enzymes having catalytic activities dictated by their microenvironment (Lai et al. 2013). MSC-derived EVs offer several advantages such as low immunogenicity, small size, off-shelf availability, and eliminate problems such as low cell survival, transmission of infections, and unpredictable behavior of the transplanted MSCs, thereby acting as a safe alternative to their parental cells (Li et al. 2019; Huňáková et al. 2020; Sharun et al. 2022). In addition, cell-free therapeutic strategies such as conditioned media and EVs overcome the limitations such as tumor differentiation and pulmonary embolization (Li et al. 2019; Sharun et al. 2022). Therefore, using MSC secretome instead of MSC improves the safety, efficacy, and affordability of cell-based therapeutics and enhances our ability to standardize the therapeutic protocol (Huňáková et al. 2020).

## 5. Conclusions

Cell-free therapeutic strategies such as conditioned media and EVs (exosomes) can potentially become the future of MSC-based therapy in managing canine osteoarthritis. However, only limited data is available to substantiate their efficacy. Therefore, *in vitro* and *in vivo* studies are needed to establish the kinetics and biodistribution of cell-free therapeutic methods such as MSC-derived EVs and secretomes in the joint environment and to identify their underlying mechanism of action. Furthermore, clinical trials are required to establish the therapeutic efficacy and safety of MSC-based cell-free therapy in clinical cases of canine osteoarthritis.

## Authors’ note

The illustration within the manuscript is created using BioRender.com.
